# Bladder leiomyoma treated with transurethral resection of bladder tumor (TURBT): Case report

**DOI:** 10.1016/j.ijscr.2022.107464

**Published:** 2022-07-30

**Authors:** Rouzy AlHalak, Sarah Alkabbani, Hala Nasseif, Neshteman Oghanna, Farhad Janahi

**Affiliations:** a 5th year medical student at Mohammed Bin Rashid University for Medicine and Health Sciences, Dubai, United Arab Emirates; b 4th year medical student at Mohammed Bin Rashid University for Medicine and Health Sciences, Dubai, United Arab Emirates; c Consultant Hispathologist/Cytopathologist, Pathology and Laboratory Medicine department, Mediclinic City Hospital, Dubai, United Arab Emirates; d Department of Urological Surgery, Mediclinic City Hospital, Dubai Healthcare City, Dubai, United Arab Emirates; e Mohammed Bin Rashid University for Medicine and Health Sciences, Dubai, United Arab Emirates

**Keywords:** Bladder leiomyoma, Transurethral resection of bladder tumor (TURBT), Urology, Case report

## Abstract

**Introduction and importance:**

Bladder leiomyomas are rare benign mesenchymal tumors, accounting only about 0.43 % of all bladder tumors. These tumors are classified based on their location, where they can be endovesical, extravesical, and intramural with the endovesical subtype being the most common. There are roughly 250 cases of bladder leiomyoma reported worldwide. In the following case report, we discuss the case of a bladder leiomyoma presenting with obstructive urinary symptoms and managed with TURBT.

**Case presentation:**

A 24-year-old female presented complaining of incomplete bladder emptying accompanied by urgency, frequency, hesitancy, and urinary incontinence. MRI and US confirmed the presence of an oval-shaped mass lesion arising from the base of the urinary bladder. Tissue biopsy and immunohistochemistry confirmed the diagnosis of bladder leiomyoma. Following confirmation of the diagnosis, the patient underwent an uneventful trans-urethral resection of bladder tumor (TURBT). During the surgery, one large bladder tumor was resected. Surgical biopsy report confirmed bladder leiomyoma.

**Clinical discussion:**

In this case report, we discuss the various management options of bladder leiomyoma and our surgical approach to this condition.

**Conclusion:**

This case highlights a rare bladder leiomyoma presenting with obstructive urinary symptoms. *Trans*-urethral resection of bladder tumor (TURBT) remains the mainstay of treatment for small, endovesical tumors. Our patient had a successful surgery with no recurrence on follow up.

## Highlights

• The importance of multidisciplinary team including pathologists in confirming the diagnosis of bladder leiomyoma.
• *Trans*-urethral resection of bladder tumor (TURBT) is a safe and un-eventful procedure to resect bladder leiomyoma.
• This is a rare case, with only 250 bladder leiomyoma cases in English literature
• TURBT remains the mainstay of treatment for small, endovesical, and easily accessible tumors.

## 1 Introduction

Bladder leiomyomas are rare benign mesenchymal tumors, accounting only about 0.43 % of all bladder tumors [1]. These tumors are classified based on their location, where they can be endovesical, extravesical, and intramural with the endovesical subtype being the most common [2]. With roughly 250 cases reported worldwide, there seems to be a preponderance of women where Goluboff et al. disclosed that women made up 76 % of the 37 cases reported [3], [4]. Bladder leiomyomas seem to predominate in the third to sixth decades of life, with a mean age of 44 years [4]. Most patients present with obstructive and irritative urinary symptoms – 49 % and 38 % respectively with a minority reporting symptoms such as hematuria and flank pain [5]. With regards to the diagnosis, Ultrasonography, CT, MRI, and cystoscopy are often performed, with MRI being the preferred method as it better assesses the site of origin, size, and boundaries of the tumor [6], [7]. Treatment is via surgical excision due to the tumor's possible growth ability and depends on the size, location, and relation to the bladder wall [4], [8]. This case report has been reported in line with the SCARE Criteria [9].

## 2 Case presentation

A 24-year-old female was referred to the urology clinic by gynecology after an incidental detection of a bladder tumor on ultrasound. At the time of the consultation, the patient was complaining of incomplete bladder emptying accompanied by urgency, frequency, hesitancy, and urinary incontinence. The patient stated that these symptoms have been present for the past 3 years, with gradual worsening over the past year. The patient's symptoms were not associated with abdominal pain, fever, hematuria, dysuria, or weight loss. On further questioning, it was revealed that the patient was otherwise healthy and not on any medications. There was no pertinent family history of malignancy or any inheritable diseases. Social history revealed that the patient is not sexually active and does not smoke or consume alcohol. Over the past four years, the patient suffered two urinary tract infections. In both incidents, the patient received oral antibiotics and did not develop any complications. However, the urinary symptoms persisted despite adequate clearance of the infection.

Past surgical history of the patient includes a laparoscopic ovarian cystectomy performed in March of 2020. Histology of the mass confirmed an endometrioma. The pelvic ultrasound performed at that time revealed a bladder tumor that necessitated further investigations.

Physical examination at the time of the urology consultation did not reveal any pertinent findings. The patient was alert, oriented, and vitally stable. There was no lymphadenopathy, suprapubic or costovertebral angle tenderness. The initial laboratory studies included a complete blood count, urinalysis, and kidney function tests - all of which reported normal findings.

**Fig. 1:**
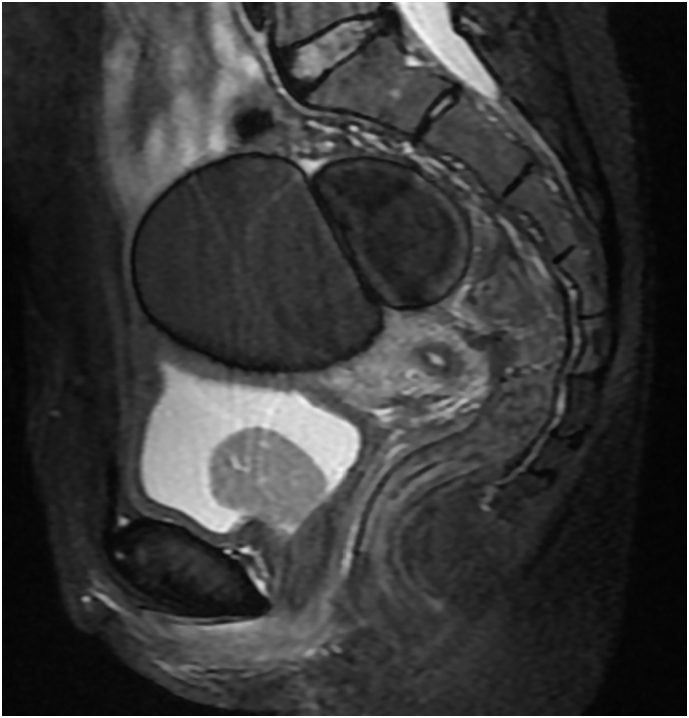
MRI showing the presence of an oval-shaped mass lesion arising from the base of the urinary bladder. The mass shows as low signal intensity on both T1 and T2 weighted images and enhances avidly following contrast medium administration.

The first pelvic ultrasound done by the gynecology team revealed a bladder tumor with mixed echogenicity. Following the ultrasound, an MRI was performed, and it confirmed the presence of an oval-shaped mass lesion arising from the base of the urinary bladder. The mass shows as low signal intensity on both T1 and T2 weighted images and enhances avidly following contrast medium administration (Fig. 1). The findings were consistent with a bladder mass.

**Fig. 2:**
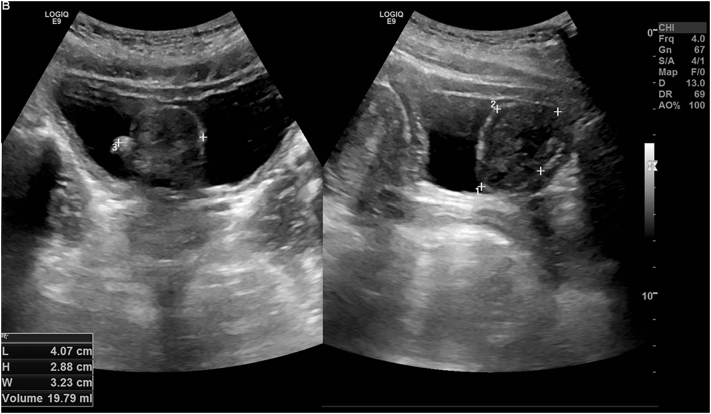
Ultrasound of the kidneys, ureters, and bladder revealing a prominent polypoidal lesion from the bladder mucosa with well-defined, encapsulated margins containing a small appendicular lobule on the surface.

An ultrasound of the kidneys, ureters, and bladder was done by the urology team in 2020, and it revealed a prominent polypoidal lesion from the bladder mucosa with well-defined, encapsulated margins containing a small appendicular lobule on the surface. The polypoidal lesion measures 4 cm × 2.8 cm × 3.2 cm with a volume of 20 cc and shows minimal flow on Doppler assessment. Appearances were suggestive of a solid lesion with well-encapsulated margins (Fig. 2).

**Fig. 3:**
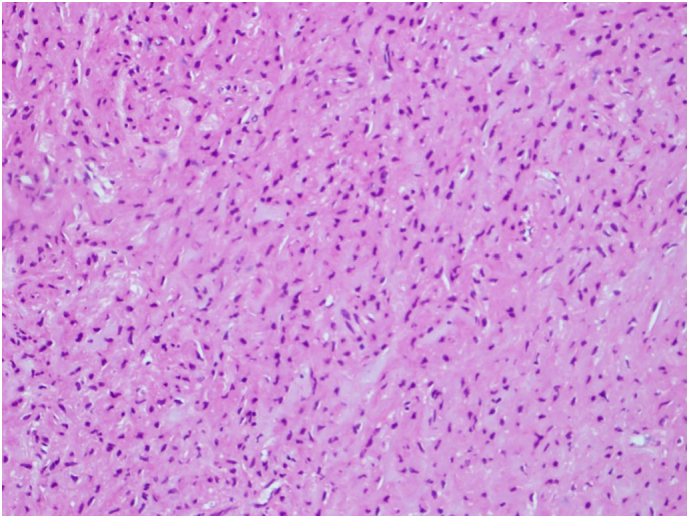
H & E stain of the mass showing epithelioid and bland spindle cells with eosinophilic cytoplasm.

**Fig. 4:**
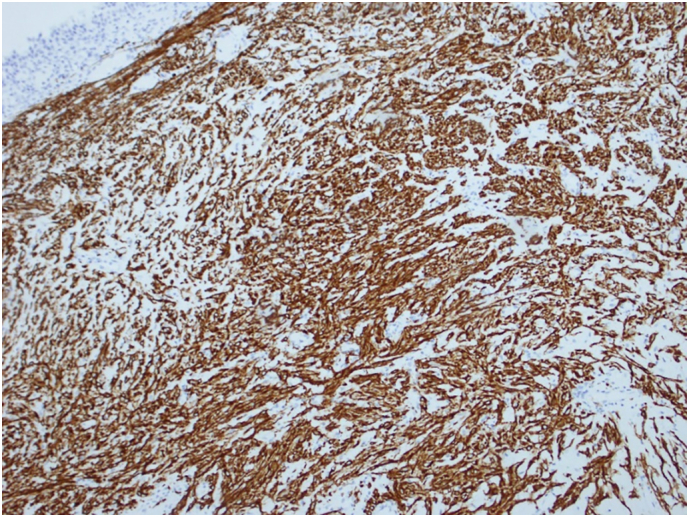
Immunohistochemistry Desmin.

**Fig. 5:**
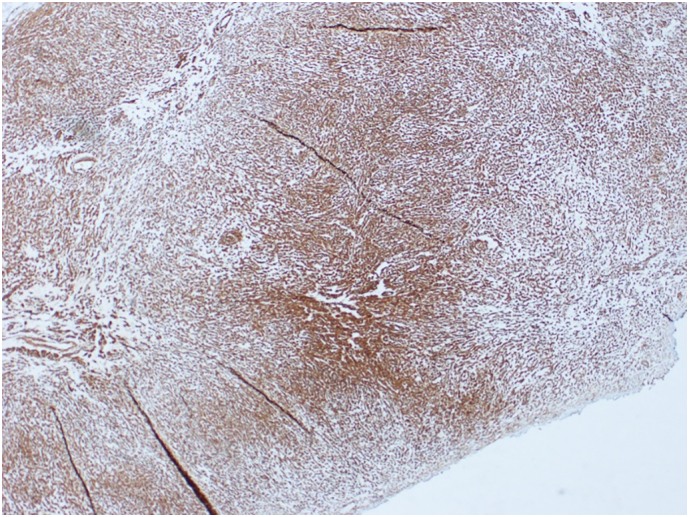
Immunohistochemistry (SMA).

The patient was concerned about the nature of this lesion and the possibility of malignancy. The results of the investigations were discussed with the patient, she was educated on the management options, and she gave an informed decision about undergoing cystoscopy and TURBT. Following that, the patient underwent cystoscopy where a large tumor was visualized at the bladder trigone. During the procedure, a tissue biopsy was taken and sent for microscopic examination. The pathological findings showed bladder tissue lined by normal urothelium with subepithelial tumor. The tumor was composed of epithelioid and bland spindle cells with eosinophilic cytoplasm. The cells displayed no nuclear pleomorphism, atypia, mitosis or necrosis (Fig. 3). On immunohistochemistry, the tumor cells showed strong expression of SMA and Desmin (Fig. 4, Fig. 5) but no expression of Cytokeratin (AE1/AE3), S100, SOX-10, CD68, ALK1, or CD117. CD117 highlights scattered mast cells between tumor cells. The appearance was suggestive of bladder leiomyoma.

Following confirmation of the diagnosis of bladder leiomyoma, the patient underwent an uneventful trans-urethral resection of bladder tumor (TURBT). The procedure was done in July 2021 by a urology consultant. During the surgery, one large bladder tumor, measuring 5 cm, was resected. A three-way 20fr foley catheter was inserted. Surgical biopsy report confirmed bladder leiomyoma. The catheter was removed in postoperative day 1. The patient tolerated the procedure well, she remained hemodynamically stable, and was discharged without any complications.

The patient was seen for follow-up one month and two months after the surgery. An ultrasound revealed no recurrence of the tumor and the patient reported that she was relieved from her urinary symptoms. The patient remains asymptomatic to this date.

## 3 Discussion and conclusion

Although rare, bladder leiomyomas are the most common mesenchymal tumors of the bladder. [2] Since there are approximately 250 cases in English literature, the epidemiology of leiomyoma is not quite clear. Review of 37 cases by Goluboff et al. found that 76 % of the bladder leiomyoma patients were females. [4] Park JW et al. reviewed 9 patients, with a mean age of 43.6 years. [10] Although bladder leiomyomas tend to occur in middle aged patients, our patient was 24 years. Chen H et al. reported a case of bladder leiomyoma in a 6-year-old boy, which is the second reported case of a pediatric bladder leiomyoma since 1966. [11]

As for clinical presentation, most patients present with obstructive urinary symptoms such as frequency, irritative symptoms, hematuria, and flank pain. [4] Some patients were asymptomatic especially when the tumor was small. Our patient presented with frequency, urgency, hesitancy, nocturia, and urinary incontinence. However, she never had hematuria when she had the bladder tumor. Since the tumor was located at the bladder trigone, it was expected that the patient would have more of obstructive symptoms.

While 62 % of the patients had open resection as treatment, recent reports have used Transurethral Resection of Bladder Tumor (TURBT) as treatment. [4] Zachariou et al. has a successful TURBT in 2020 without recurrence on 12 months post operation. [12] Despite thorough removal of the trigone wall, Zachariou et al. reported no scarring in the bladder trigone or distortion of the ureteral orifices. The advantage that Goluboff et al. found with open surgery is that none required a second procedure, while 18 % of patients who underwent TURBT required a second procedure. [4]

To our current knowledge, TURBT remains the mainstay of treatment for small, endovesical, and easily accessible tumors. [3] In the case of an unfavorable position or a huge tumor, other surgical options are available such as segmental resection, laparoscopic partial cystectomy, or cystoprostatectomy. [3] Lyons et al. removed a large transmural extravesical bladder leiomyoma by laparoscopy. [13] Similarly, Al Solumany et al. performed an open partial cystectomy on a bladder submucosal mass in the anterolateral wall. Due to its huge size (7x5x4 cm), open partial cystectomy was required, and was successful. [14] Outpatient transurethral laser ablation (TULA) using 1470 nm diode laser is time-efficient procedure and low risk of bladder perforation. [15] The role of TULA has also been covered in the NICE guidelines for treatment of bladder cancer. [16]

In conclusion, since our patient had a small endovesical tumor at the bladder neck, TURBT was successful in resecting the tumor without recurrence. Follow up is important to ensure symptom resolution and no recurrence of bladder tumor. This case report demonstrates a rare case of bladder leiomyoma where TURBT was employed without recurrence of the tumor. We highlight the importance of multidisciplinary team approach in providing patient-centered care.

## Provenance and peer review

Not commissioned, externally peer-reviewed.

## Sources of funding

No funding.

## Ethical approval

N/A.

## Consent

Obtained.

## Research registration

N/A.

## Guarantor

Farhad Janahi.

## CRediT authorship contribution statement

Rouzy AlHalak: Contributed to the conception and design of the study, drafted the article and critically revised it for important intellectual content, final approval of the version to be submitted.

Sarah AlKabbani: Contributed to the conception and design of the study, drafted the article

Hala Nasseif: Contributed to the conception and design of the study, drafted the article

Neshteman Oghanna: Contributed to the conception and design of the study

Farhad Janahi: critically revised the manuscript for important intellectual content, gave final approval for the study to be submitted.

## Declaration of competing interest

None.
